# Impact of COVID-19 on epidemic trend of hepatitis C in Henan Province assessed by interrupted time series analysis

**DOI:** 10.1186/s12879-023-08635-9

**Published:** 2023-10-17

**Authors:** Yanyan Li, Xinxiao Li, Xianxiang Lan, Chenlu Xue, Bingjie Zhang, YongBin Wang

**Affiliations:** Department of Epidemiology and Health Statistics, School of Public Health, Xinxiang Medical University, Xinxiang, 453000 Henan Province People’s Republic of China

**Keywords:** Interruption time series analysis, Autoregressive comprehensive moving average model, COVID-19, Hepatitis C, Intervention analysis

## Abstract

**Objective:**

Hepatitis C presents a profound global health challenge. The impact of COVID-19 on hepatitis C, however, remain uncertain. This study aimed to ascertain the influence of COVID-19 on the hepatitis C epidemic trend in Henan Province.

**Methods:**

We collated the number of monthly diagnosed cases in Henan Province from January 2013 to September 2022. Upon detailing the overarching epidemiological characteristics, the interrupted time series (ITS) analysis using autoregressive integrated moving average (ARIMA) models was employed to estimate the hepatitis C diagnosis rate pre and post the COVID-19 emergence. In addition, we also discussed the model selection process, test model fitting, and result interpretation.

**Results:**

Between January 2013 and September 2022, a total of 267,968 hepatitis C cases were diagnosed. The yearly average diagnosis rate stood at 2.42/100,000 persons. While 2013 witnessed the peak diagnosis rate at 2.97/100,000 persons, 2020 reported the least at 1.7/100,000 persons. The monthly mean hepatitis C diagnosed numbers culminated in 2291 cases. The optimal ARIMA model chosen was ARIMA (0,1,1) (0,1,1)_12_ with AIC = 1459.58, AICc = 1460.19, and BIC = 1472.8; having coefficients MA1=-0.62 (t=-8.06, *P* < 0.001) and SMA1=-0.79 (t=-6.76, *P* < 0.001). The final model’s projected step change was − 800.0 (95% confidence interval [*CI*] -1179.9 ~ -420.1, *P* < 0.05) and pulse change was 463.40 (95% *CI* 191.7 ~ 735.1, *P* < 0.05) per month.

**Conclusion:**

The measures undertaken to curtail COVID-19 led to a diminishing trend in the diagnosis rate of hepatitis C. The ARIMA model is a useful tool for evaluating the impact of large-scale interventions, because it can explain potential trends, autocorrelation, and seasonality, and allow for flexible modeling of different types of impacts.

**Supplementary Information:**

The online version contains supplementary material available at 10.1186/s12879-023-08635-9.

## Introduction

Hepatitis C is a viral infection caused by the hepatitis C virus (HCV). Symptoms commonly include decreased appetite, nausea, and general weakness, which can progress to liver failure and brain tissue accumulation, posing significant health risks and public health concerns. The World Health Organization (WHO) estimates that HCV infection has become a substantial global public health burden, affecting approximately 3% of the population worldwide [1]. Hepatitis C is the leading cause of chronic liver disease among over 170 million individuals globally [2]. The disease manifests in a wide range of severity, from mild self-limiting cases to cirrhosis and hepatocellular carcinoma [3]. Regrettably, no vaccine is currently available for hepatitis C. Chronic hepatitis C is highly prevalent and associated with an increased risk of progressive liver fibrosis, ultimately leading to cirrhosis and liver failure [4]. However, significant progress has been made with the development of direct-acting antiviral (DAA) medications. Notably, the combination of sofosbuvir and velpatasvir, together with sofosbuvir and ledipasvir, have proven highly successful in clinical trials and are now considered the cornerstone of HCV treatment [5]. Despite these advancements, the global mission to eradicate HCV faces challenges, such as the high cost of DAA medications and the absence of a vaccine. Effective management of hepatitis C remains a complex task due to its widespread prevalence and associated risk factors [5].

The emergence of COVID-19 as a novel coronavirus has placed immense strain on global healthcare systems. Early reports from China highlighted the overwhelming pressure faced by hospital staff during the initial stages of the pandemic[6]. The healthcare infrastructure in many countries has been severely disrupted due to the high demand for COVID-19 care and the reallocation of resources. As a result, the efforts to eliminate hepatitis C, including screening, diagnosis, and treatment, have been significantly reduced or even halted. This poses significant challenges to the WHO’s goal of eradicating hepatitis C, both in terms of screening and treatment. The Chinese CDC has issued guidelines to postpone non-essential procedures and routine outpatient visits in order to alleviate the burden on the healthcare system caused by COVID-19. The pandemic has not only impeded hepatitis eradication plans, but also led to a decrease in routine HCV antibody screening, clinical care, and treatment opportunities in the first half of 2020 [7].

Furthermore, the implementation of social distancing measures, lockdowns, isolation protocols, and the perceived risk of contracting SARS-CoV-2 during hospital visits have resulted in fewer visits for non-COVID-19 related illnesses. This delay in seeking medical care may hinder the early detection and treatment of hepatitis C, thereby increasing the health risks for individuals affected by the disease [8]. The delayed identification and subsequent treatment of hepatitis C-infected individuals may also contribute to a higher likelihood of virus transmission and an increased risk of disease progression in untreated individuals. This can lead to the development of advanced liver disease and potential transmission of the virus [9]. It is estimated that a one-year delay in the diagnosis and treatment of hepatitis could result in 44,800 new cases of liver cancer and 72,300 deaths from hepatitis C worldwide by 2030 [9]. Given the global commitment to eliminate hepatitis by 2030, it is crucial to prioritize hepatitis planning as soon as circumstances permit [10].

In this study, an interrupted time series (ITS) analysis was employed to assess the influence of COVID-19 on hepatitis C diagnosis rate, contributing a solid scientific foundation for enhancing hepatitis C prevention and control strategies in Henan Province.

## Materials and methods

**Fig. 1:**
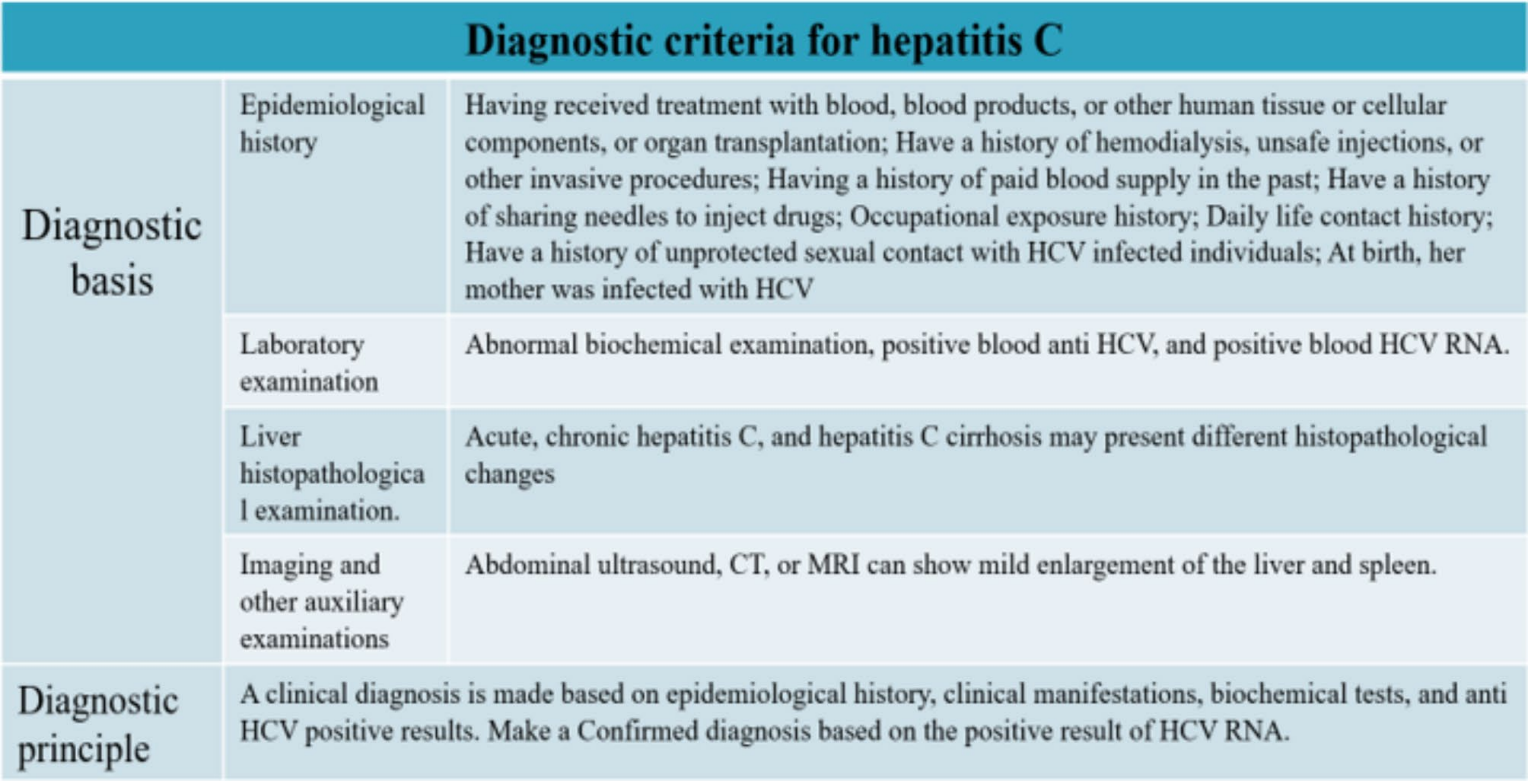
Diagnostic criteria for hepatitis C

The data used in this study originates from hepatitis C diagnosis cases reported by the Henan Provincial Health Commission spanning from January 2013 to September 2022. The diagnosis data of hepatitis C is defined in accordance with the diagnostic criteria specified by the Henan Provincial Health Commission. Figure 1 illustrates the diagnostic criteria for hepatitis C.

The interrupted time series (ITS) analysis serves to assess the impact of interventions by comparing indicators within the time series both before and after the intervention point. The two primary models commonly utilized for this analysis are the ARIMA and Segmented Regression Model (SRM). In cases where the trend of dependent variables displays non-linearity with strong seasonality and periodicity, as often observed in time series data, a direct application of the SRM could compromise the accuracy of intervention effect evaluation. Instead, ARIMA may be employed for a more appropriate analysis [11].

### ARIMA modeling process

The constructed ARIMA model is represented as ARIMA (p, d, q) (P, D, Q) s. Here, p signifies the autoregressive (AR) orders, q represents the moving average (MA) orders, and d stands for the number of differences applied to make the time series stationary [12]. D indicates the degree of seasonal difference, while P and Q denote the AR and MA terms of the seasonal components [13]. The ARIMA modeling process encompasses the following steps: (1) Data smoothing: Initially, the stationarity of the time series data is assessed. For non-stationary time series, iterative differencing is conducted until stationarity is achieved [13]. If the time series has seasonal factors, the seasonal differencing is employed to control or eliminate the impact of seasonal autocorrelation. (2) Model identification: The “auto.arima()” function within the forecast package automatically tests various order combinations and selects the optimal model, aiding in the determination of appropriate orders. (3) Model selection: Based on model selection criteria, the model is evaluated using the Akaike Information Criterion (AIC) and Bayesian Information Criterion (BIC) to identify the most suitable one. (4) Model validation: Residuals of the chosen model are examined to determine if they exhibit characteristics of white noise. Autocorrelation testing is carried out by analyzing residual plots and performing the Ljung-Box *Q* test.

### Use ITS-ARIMA model to evaluate interventions

ITS analysis assesses the impact of intervention measures by evaluating their influence on specific outcomes. Three primary types of intervention effects include step change, pulse change, and slope change [13]. Predefining the expected shape of the intervention effect is essential, which is influenced by the intervention’s nature (temporary or continuous) and the evaluation’s outcomes. Combinations of influencing variables are often utilized to express changes, like step change and slope/pulse change or step change and pulse change. It is important to account for potential delays in impact, specifying a reasonable time frame to observe the effects based on prior research to avoid spurious correlations [14]. When necessary, controlling and mitigating the impact’s delay on the time series may involve excluding data during the intervention’s transitional period. By comparing estimated indicator values post-intervention with values assumed under no intervention, the researcher calculates the intervention’s impact at specific time points. These effects can be modeled effectively through step and pulse functions or straight lines with unit slopes [14]. Once the final model is selected, the intervention’s impact can be estimated accurately.

## Results

A total of 267,968 cases of hepatitis C were diagnosed between January 2013 and September 2022, resulting in an annual average diagnosis rate of 2.42 per 100,000 people. The highest diagnosis rate occurred in 2013 at 2.97 per 100,000 people, while the lowest was recorded in 2020 at 1.7 per 100,000 people. The average monthly diagnosis cases of hepatitis C was 2291.

**Fig. 2:**
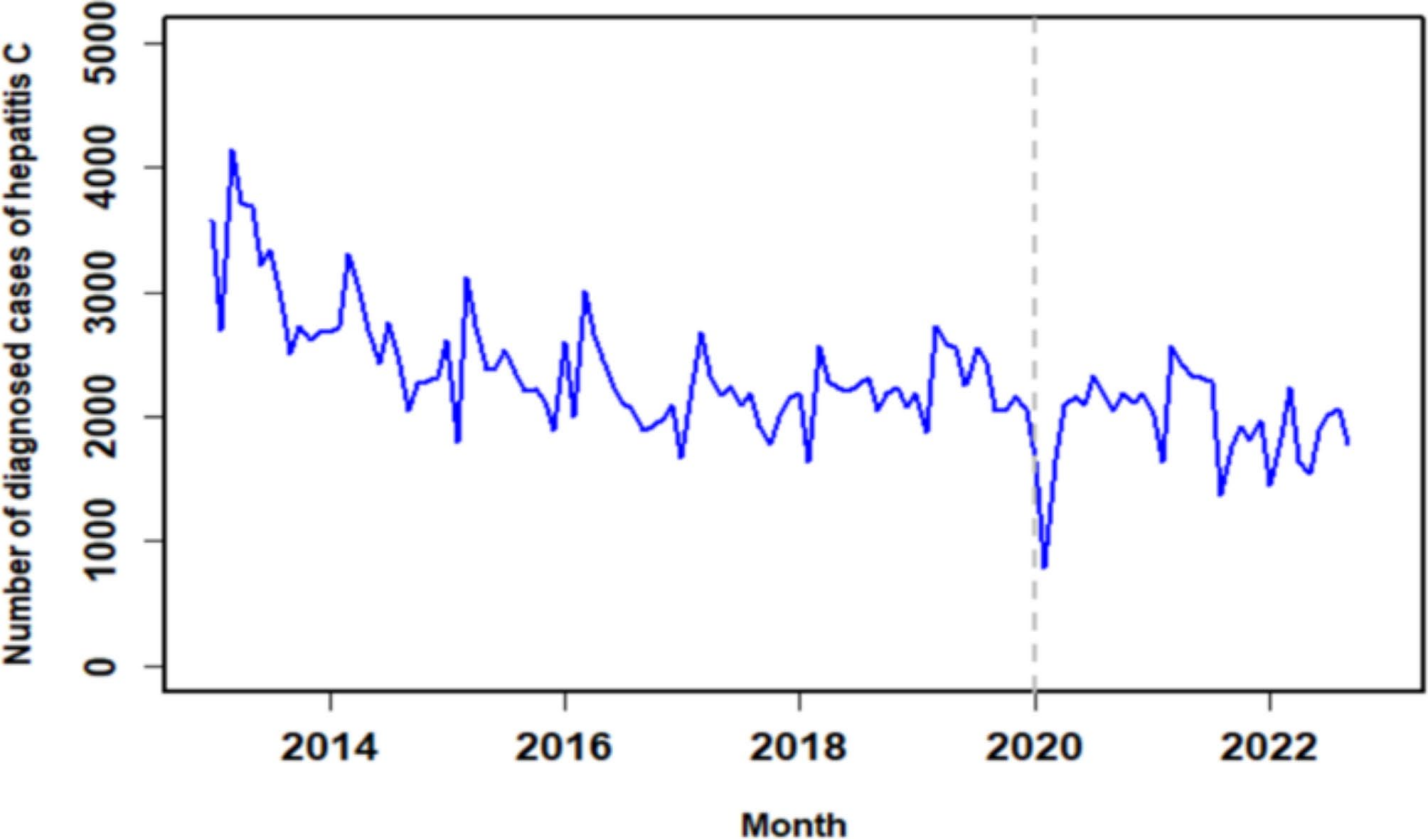
Henan Province Hepatitis C Time Series Plot

The diagnosis rate for hepatitis C was analyzed using the ARIMA model. Figure 2 displays a time series analysis of hepatitis C diagnosis rate in Henan spanning from January 2013 to September 2022. The data is divided into two segments: pre-COVID-19 (from January 2013 to December 2019) and post-COVID-19 (from January 2020 to September 2022). The insights gained from Fig. 2 highlight a noticeable decrease in hepatitis C diagnosis rate during the COVID-19 outbreak in 2020, followed by a subsequent increase. Across the broader timeframe from 2013 to 2022, the diagnosis series of hepatitis C exhibited cyclic patterns. Through the application of differencing to adjust for this trend and periodicity, the hepatitis C diagnosis series demonstrated enhanced stability.

The Box-Cox method was employed, along with the “auto.arima” function in the R software, to fit the hepatitis C diagnosis series in Henan Province from January 2013 to September 2022. ACF and PACF plots are depicted in Supplementary Fig.S1. In this illustration, bars above or below the dotted line represent statistically significant autocorrelation (*P* < 0.05). Both ACF and PACF plots (Supplementary Fig.S1a) reveal undifferentiated autocorrelation and partial autocorrelation patterns, with noticeable significant autocorrelation. Supplementary Fig.S1b presents autocorrelation and partial autocorrelation post-differencing. In comparison to Supplementary Fig.S1a, differencing effectively removed much of the autocorrelation.

The ARIMA (0,1,1) (0,1,1)_12_ model yielded the lowest AIC (1459.58), AICc (1460.19), and BIC (1472.8). Consequently, this model structure was selected as the optimal. Diagnostic results indicated that for the ARIMA (0,1,1) (0,1,1)_12_ model, MA1=-0.62 (t=-8.06, *P* < 0.001) and SMA1=-0.79 (t=-6.76, *P* < 0.001); ACF and PACF plots of residuals indicated most correlation coefficients were within the confidence interval (Supplementary Fig.S2). The Ljung-Box *Q* test results demonstrated no statistically significant difference among residuals for different lag periods (*P* = 0.408), affirming that model residuals constituted white noise. These results validate the ARIMA (0,1,1) (0,1,1)_12_ model.

Over time, the time series plot displayed relatively constant variance. The histogram of the time series showed normally distributed prediction errors, with the mean adhering to normal distribution as well. Residuals showcased no discernible pattern or significant autocorrelation, and they followed a normal distribution. The Ljung-Box *Q* test yielded a *P*-value of 0.408, confirming the chosen model’s good fit.

**Fig. 3:**
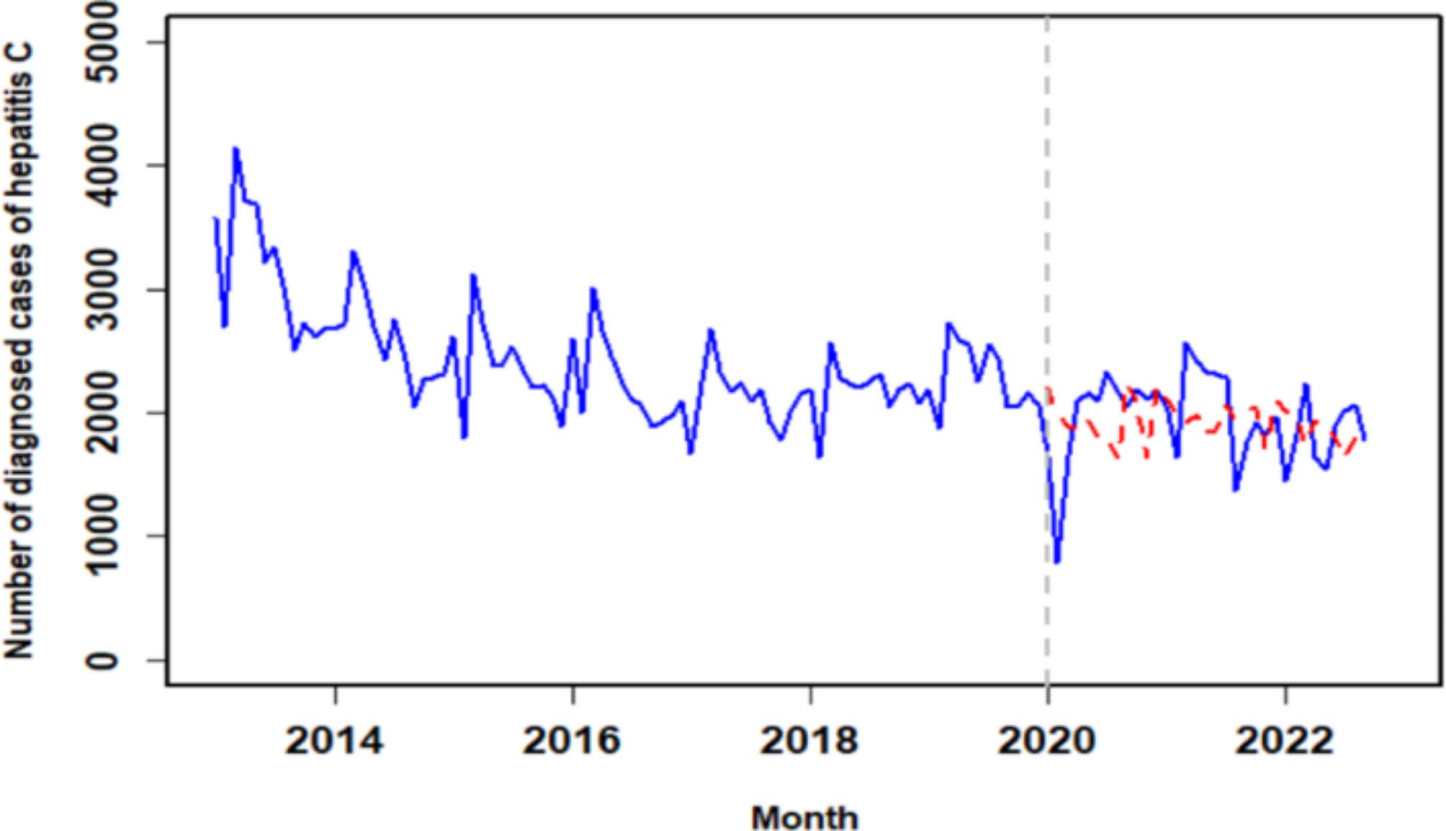
Observed and predicted values without intervention based on ITS-ARIMA model

The final model estimated a step change of -800.0 (95% *CI* -1179.9 ~ -420.1, *P* < 0.05) and a pulse change of 463.40 (95% *CI* 191.7 ~ 735.1, *P* < 0.05) per month. Figure 3; Table 1 depict the comparison between predicted and observed values of the ARIMA model without intervention (counterfactual).

From January 2013 to September 2022, an analysis was conducted on the fitting and observed values of hepatitis C diagnosis cases both before and after the onset of COVID-19. The findings suggested a decrease in hepatitis C diagnosis cases in January 2020, with an anticipation of the impact being transient. Since January 2020, the influence of COVID-19 on hepatitis C diagnosis rate was modeled using a pulse function. Following the emergence of COVID-19, a decline in hepatitis C diagnosis cases was observed. A step function was utilized to fit potential long-term fluctuations in the number of hepatitis C diagnosis cases. The final model revealed a sudden reduction in diagnosis cases post the onset of COVID-19, followed by a gradual increase back to pre-COVID-19 levels. Given the nature of the intervention, we assumed an immediate decrease in diagnosis cases post-intervention (step change) and an accompanying pulse change. Hence, variables representing both types of impacts were incorporated into the model.

Moreover, when comparing the ITS-ARIMA model with the BSTS model (Fig. 3), results indicated that the mean absolute percentage error (MAPE = 19.95%) of the ITS-ARIMA model’s predictions was lower than that of the BSTS model (MAPE = 25.7%). This implies that the prediction performance of the ITS-ARIMA model surpassed that of the BSTS model, demonstrating the former’s superior predictive capabilities.

**Table 1:** Prediction of Hepatitis C diagnosis cases from January 2020 to September 2022 after COVID-19 intervention by ITS-ARIMA model and BSTS model

Time	Observed data	ITS-ARIMA model	BSTS model
2020-01	1659	2203	1955
2020-02	785	2009	2849
2020-03	1662	1886	2572
2020-04	2100	1881	2457
2020-05	2159	1946	2268
2020-06	2090	1790	2379
2020-07	2326	1804	2302
2020-08	2180	1645	2003
2020-09	2053	2232	2068
2020-10	2196	2107	2133
2020-11	2106	1634	2100
2020-12	2188	2221	2239
2021-01	2022	2099	1939
2021-02	1644	1997	2871
2021-03	2569	1925	2552
2021-04	2407	1979	2466
2021-05	2325	1851	2279
2021-06	2310	1866	2389
2021-07	2285	2070	2294
2021-08	1364	1935	1998
2021-09	1760	2040	2085
2021-10	1920	2026	2143
2021-11	1818	1723	2117
2021-12	1964	2093	2254
2022-01	1455	2011	1971
2022-02	1828	1956	2879
2022-03	2238	1754	2569
2022-04	1635	1932	2474
2022-05	1551	1892	2305
2022-06	1895	1793	2396
2022-07	2015	1636	2276
2022-08	2069	1794	2014
2022-09	1788	1810	2090

## Discussion

Throughout the period from 2013 to 2020, the diagnosis rate of hepatitis C displayed a declining trend following the onset of the COVID-19 pandemic, particularly evident in early 2020. As COVID-19 emerged, Henan Province implemented emergency response measures to curb its spread, which subsequently affected hepatitis C diagnosis rate.

Table 1 provides a comparison of predicted and actual values under a scenario without the COVID-19 pandemic (counterfactual). At the beginning of 2020, the actual diagnosis rate was considerably lower than the predicted value. Findings suggest that hepatitis C diagnosis rate in Henan Province experienced a decrease due to COVID-19 prevention measures, consistent with European research outcomes [8]. The decline in hepatitis C diagnosis cases can be attributed to two primary factors [15].First, long-standing effective measures for hepatitis C contributed significantly, including continuous efforts by the Chinese government like robust monitoring systems, increased budgets, and efficient control and treatment. Second, the impact of COVID-19 played a role. There may be several reasons for the decline of hepatitis C diagnosis rate caused by the COVID-19 [16–20]. First, the COVID-19 crisis caused most of the medical resources to be invested in the treatment of COVID-19 patients. Second, the impact of COVID-19 on the elimination of HCV included the redistribution of medical resources and the interruption of treatment. Third, the unwillingness of Hepatitis C patients to seek medical treatment for fear of COVID-19 infection. Fourth, the follow-up plan of Hepatitis C patients could not be implemented owing to the suspension of the medical center, which led to the reduction of the diagnosis rate and treatment rate of Hepatitis C. Fifth, to reduce hospital congestion, it is not recommended for patients with chronic diseases or symptoms to seek medical assistance. Sixth, the opening hours of health facilities are shortened, and the ability of the elderly or those with limited mobility to pay for care or transportation costs is reduced. Individual hepatitis C patients find it difficult to access medical services. Seventh, people with HCV infection may be reluctant to have a medical check-up due to the strict prevention and control measures and the mandatory requirement for the negative results of nucleic acid testing. Eighth, during the lockdown period, under-reporting or delayed reporting may be inevitable for a passive monitoring system.

In general, from 2013 to 2020, the HCV diagnosis rate seems to be declining. As can be seen from Fig. 3, the COVID-19 pandemic will begin to decline sharply in 2020, and then the HCV diagnosis rate will return to the previous level. This trend can be attributed to the relaxation of COVID-19 policy. The redistribution of medical resources and changes in medical demand may affect the diagnosis rate, making it return to the level before COVID-19 [21]. These findings align with Qin Zhou’s research utilizing ITS analysis to evaluate COVID-19’s impact on infectious diseases, including hepatitis C [21]. Through sensitivity analysis, the ITS-ARIMA model demonstrated superior predictive performance compared to the BSTS model, confirming the former’s reliability.

It is essential to educate hepatitis C patients, especially those with cirrhosis or advanced liver disease, about COVID-19 risks and preventive measures, given the potential severity of COVID-19 in such cases [22]. The global aim of eliminating hepatitis C by 2030 has been hindered by the COVID-19 pandemic [10]. Although Henan Province implemented preventive measures against COVID-19 early on, the pandemic negatively impacted chronic disease patient care. The postponement of non-emergency healthcare services strained the healthcare system. Delayed identification of HCV-infected individuals increases transmission risks [23]. Telemedicine offers a solution for patient follow-ups, safeguarding patients and medical staff from COVID-19 transmission [24]. However, its expanded use also introduces security concerns, necessitating ongoing improvements.

This study’s strength lies in its utilization of Henan Province’s notifiable infectious disease data to analyze COVID-19’s impact on hepatitis C diagnosis rate, controlling for seasonal and cyclical effects. However, the post-COVID-19 data collection cycle is relatively short, potentially leading to delays, inaccuracies, and data omissions. Besides, the data in the study is diagnosis cases for hepatitis C that has been legally reported, and is not the actual incidence data for hepatitis C. Rapid policy changes further affect the study’s scope, warranting future research.

## Conclusion

Under the influence of COVID-19 prevention measures, hepatitis C diagnosis rate in Henan Province experienced a downward trend, possibly linked to these preventative efforts. The disease exhibited cyclical and seasonal traits. The ARIMA model effectively explained trends, autocorrelation, and seasonality, offering flexibility in understanding intervention impacts. Balancing COVID-19 prevention with hepatitis C management is crucial.

### Electronic supplementary material

Below is the link to the electronic supplementary material.

Supplementary Material 1

Supplementary Material 2

## Data Availability

Hepatitis C data obtained from Henan Provincial Health Commission (https://wsjkw.henan.gov.cn/zfxxgk/yqxx/index.html).
