# Supplementary figures and images for: Impact of COVID-19 on epidemic trend of hepatitis C in Henan Province assessed by interrupted time series analysis
Source: BMC Infect Dis. 2023 Oct 17;23:691. doi: 10.1186/s12879-023-08635-9 (PMC10580576; doi:10.1186/s12879-023-08635-9)

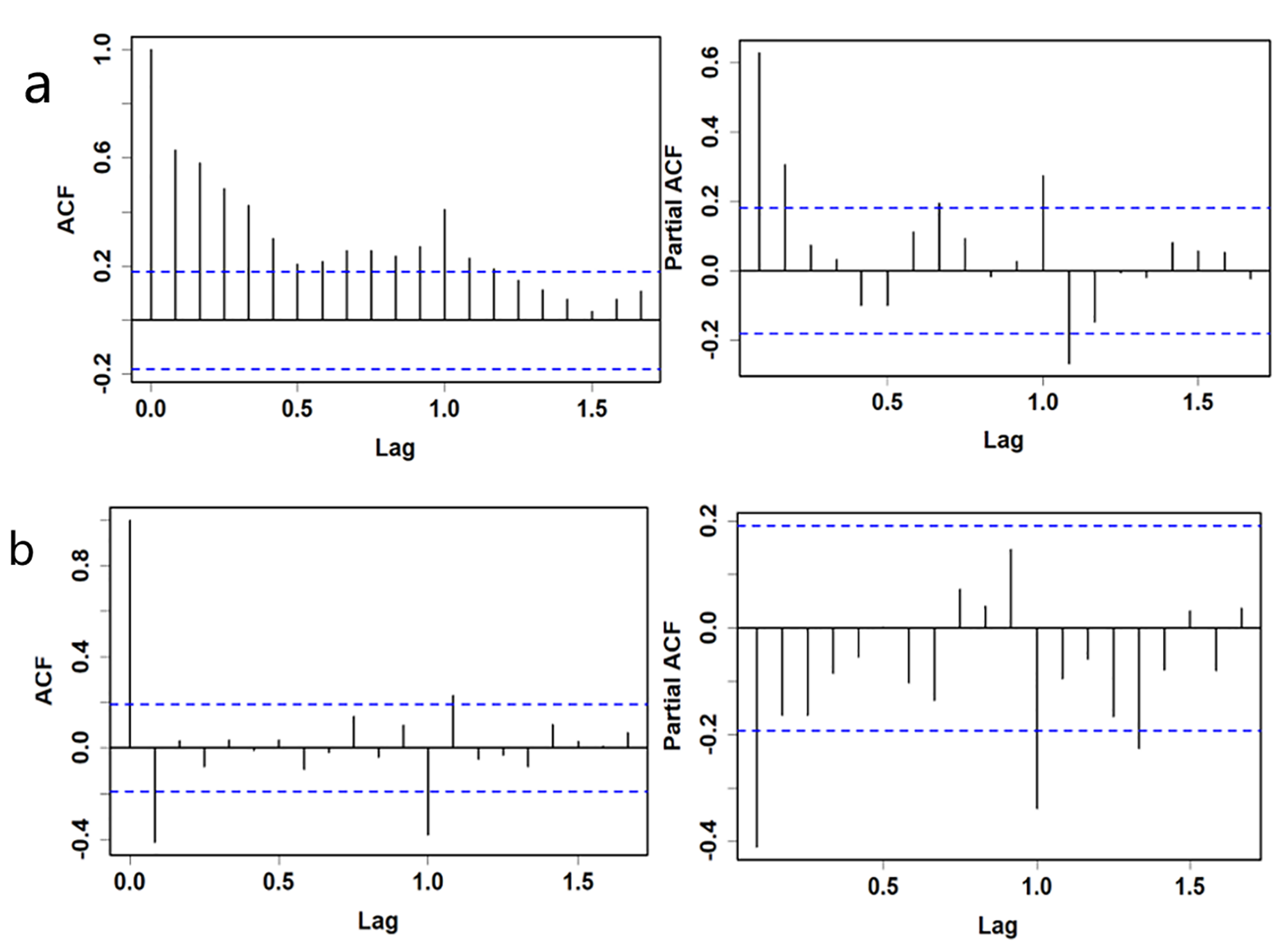

Supplement: Supplementary file 1 — Supplementary Material 1 [file 12879_2023_8635_MOESM1_ESM.tif]

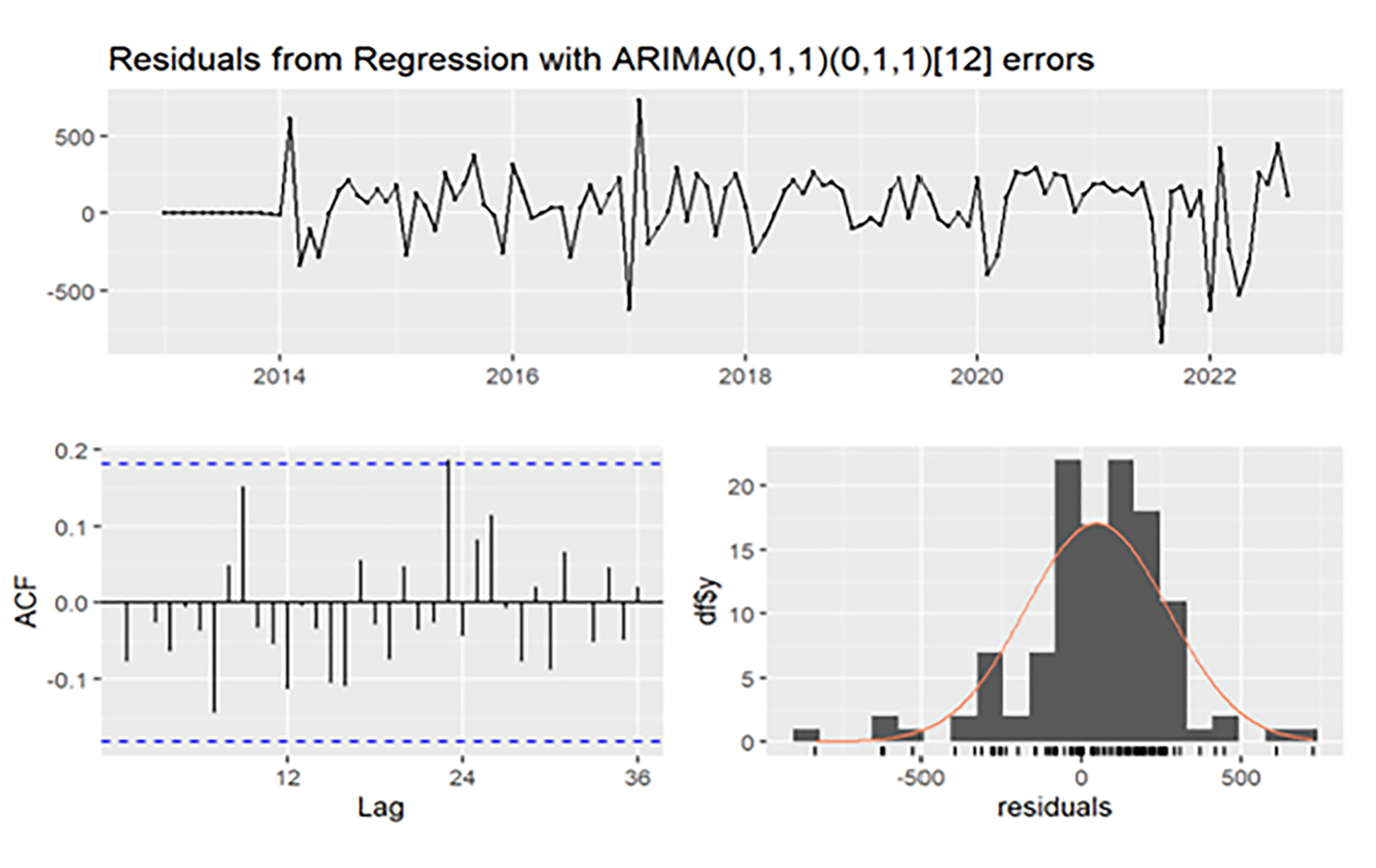

Supplement: Supplementary file 2 — Supplementary Material 2 [file 12879_2023_8635_MOESM2_ESM.tif]
